# Interpreting antidepressant prescribing trends in Croatia: a letter to the editor

**DOI:** 10.3325/cmj.2026.67.324

**Published:** 2026-08

**Authors:** Hiba Zeeshan, Muhammad Faisal Shabbir, Inshira Noor

**Affiliations:** Department of Medicine, Liaquat University of Medical and Health Sciences, Jamshoro, Pakistan

To the editor:

We read with great interest the article by Gvozdanović et al (1) on antidepressant prescription patterns in the Croatian population. The authors should be commended for examining such an important topic and highlighting the need for targeted and evidence-based health policies. However, we would like to point out an important overinterpretation regarding the prescription trends during the COVID-19 pandemic. The authors stated that the sudden rise in prescriptions in March 2020 may reflect “stockpiling.” Although stockpiling did occur in European regions such as Scandinavia, France, and Northern Ireland, these findings may not necessarily be generalizable to Croatia without direct evidence. Furthermore, the authors attributed the increased use of fluvoxamine to its ability to reduce COVID-19 hospitalization rates without empirically testing this assumption within the data set. Research from Croatia did not demonstrate reduced hospitalization among patients prescribed fluvoxamine during the pandemic (2), making this explanation insufficiently supported by the available data. Moreover, an interrupted time-series (ITS) by Selke Krulichová et al, cited by the authors, found that the changes in antidepressant use due to the pandemic were minor fluctuations (3). Although the authors appropriately hedged by stating that “the trend cannot be simply attributed to the pandemic, but its potential influence cannot be ignored,” the claim is inconsistent with their attributions, which appear stronger than the evidence presented in both their data set and the cited ITS study. Additionally, the proposed explanation that Croatia lags behind European countries in rising antidepressant use due to high benzodiazepine consumption is not supported by evidence, as no data are available for benzodiazepine co-prescribing, switching, and substitution at the patient level. Attributing lower antidepressant use to high benzodiazepine consumption risks constituting an ecological fallacy. A correlation between national anxiolytic use and regional antidepressant use does not prove substitution. Despite these limitations, the study is an important contribution to understanding antidepressant patterns in Croatia. We would recommend that the authors use the ITS methodology in future studies to evaluate pandemic-related changes and employ patient-level linkage of benzodiazepine and antidepressant prescriptions.

1 GvozdanovićK MužićR OrehovačkiH Štimac GrbićD Antidepressant prescribing pattern in Croatia: a retrospective, longitudinal study from 2017 to 2022. Croat Med J 2026 67 55 65 10.3325/cmj.2026.67.55 42104645 PMC13176931

2 TrkuljaV KodvanjI Outpatients prescribed with fluvoxamine around the time of COVID-19 diagnosis are not at a reduced risk of subsequent hospitalization and death compared to their non-prescribed peers: population-based matched cohort study. Eur J Clin Pharmacol 2023 79 643 55 10.1007/s00228-023-03479-3 36961578 PMC10036980

3 Selke KrulichováI HallbergA SelkeGW AaltonenK CasulaM FürstJ  Impact of the COVID-19 pandemic on antidepressant use in eleven European regions: a comparative time series analysis 2018-2022. Soc Psychiatry Psychiatr Epidemiol 2026 61 701 12 10.1007/s00127-025-02962-9 40696201 PMC13021844

